# Decrease of CA 19–9 during chemotherapy with gemcitabine predicts survival time in patients with advanced pancreatic cancer

**DOI:** 10.1054/bjoc.1999.1035

**Published:** 2000-02-01

**Authors:** U Halm, T Schumann, I Schiefke, H Witzigmann, J Mössner, V Keim

**Affiliations:** Departments of Internal Medicine II and Surgery II, University of Leipzig, Philipp-Rosenthal-Strasse 27, Leipzig, D-04103, Germany

**Keywords:** pancreatic cancer, chemotherapy, CA 19–9, gemcitabine

## Abstract

Chemotherapy with gemcitabine has been shown to be an effective regimen in advanced or metastatic pancreatic cancer with improvement of both quality of life and survival time. The response of the tumour marker CA 19–9 to chemotherapy with gemcitabine was studied in order to find out whether it is related to survival time of patients. Forty-three consecutive patients (median age 61 years, range 39–76 years; 20 males, 23 females) suffering from histologically proven locally advanced or metastatic pancreatic adenocarcinoma and a baseline Karnofsky-index ≥ 60 were treated with gemcitabine in a dose of 1000 mg m^−2^weekly × 7 followed by 1 week of rest during the first cycle and thereafter 1000 mg m^−2^weekly × 3 followed by 1 week of rest until progression. In 36 of 43 patients serial measurements of CA 19–9 could be performed. Patients with a decrease of > 20% of the baseline CA 19–9 level after 8 weeks of treatment (*n* = 25) had a significantly better median survival than patients with a rise or a decrease ≤ 20% (*n* = 11) (268 vs 110 days;*P*< 0.001). The response of CA 19–9 was the strongest independent predictor of survival (*P*< 0.001) in the multivariate analysis. In conclusion, a decrease of CA 19–9 > 20% during the first weeks of chemotherapy with gemcitabine is associated with a better survival of patients with locally advanced or metastatic pancreatic cancer. Serial measurements of CA 19–9 are useful to decide whether further chemotherapy after the first weeks of treatment is indicated. © 2000 Cancer Research Campaign


British Journal of Cancer (2000) 82(5), 1013–1016
© 2000 Cancer Research Campaign
DOI: 10.1054/ bjoc.1999.1035, available online at http://www.idealibrary.com on IDEAL®

### Decrease of CA 19–9 during chemotherapy with gemcitabine predicts survival time in patients with advanced pancreatic cancer

**U Halm[1], T Schumann[1], I Schiefke[1], H Witzigmann[2], J Mössner[1] and V Keim[1]**

Departments of [1]Internal Medicine II and [2]Surgery II, University of Leipzig, Philipp-Rosenthal-Strasse 27, D-04103 Leipzig, Germany

**Summary** Chemotherapy with gemcitabine has been shown to be an effective regimen in advanced or metastatic pancreatic cancer with improvement of both quality of life and survival time. The response of the tumour marker CA 19–9 to chemotherapy with gemcitabine was studied in order to find out whether it is related to survival time of patients. Forty-three consecutive patients (median age 61 years, range 39–76 years; 20 males, 23 females) suffering from histologically proven locally advanced or metastatic pancreatic adenocarcinoma and a baseline Karnofsky-index ≥ 60 were treated with gemcitabine in a dose of 1000 mg $m^{-2}$ weekly × 7 followed by 1 week of rest during the first cycle and thereafter 1000 mg $m^{-2}$ weekly × 3 followed by 1 week of rest until progression. In 36 of 43 patients serial measurements of CA 19–9 could be performed. Patients with a decrease of > 20% of the baseline CA 19–9 level after 8 weeks of treatment ($n$ = 25) had a significantly better median survival than patients with a rise or a decrease ≤ 20% ($n$ = 11) (268 vs 110 days; $P < 0.001$). The response of CA 19–9 was the strongest independent predictor of survival ($P < 0.001$) in the multivariate analysis. In conclusion, a decrease of CA 19–9 > 20% during the first weeks of chemotherapy with gemcitabine is associated with a better survival of patients with locally advanced or metastatic pancreatic cancer. Serial measurements of CA 19–9 are useful to decide whether further chemotherapy after the first weeks of treatment is indicated. © 2000 Cancer Research Campaign

**Keywords**: pancreatic cancer; chemotherapy; CA 19–9; gemcitabine

Most patients with adenocarcinoma of the pancreas present locally advanced or metastatic disease at the time of diagnosis. With only about 1% of patients still alive 5 years from the time of diagnosis, these patients have a very poor prognosis. Gemcitabine (2′,2′-difluorodeoxycytidine), a nucleoside analogue with a mild toxicity profile (Aapro et al, 1998), has been shown to improve both clinical benefit and survival in patients with advanced pancreatic cancer compared to treatment with 5-fluorouracil, although overall survival was poor with a median survival of less than 6 months (Burris et al, 1997). To identify any subgroups of patients in which chemotherapy with gemcitabine improves survival, prognostic parameters during the first cycles of chemotherapy would be helpful. Unfortunately, assessment of tumour diameters by imaging techniques to monitor response to chemotherapy are often inaccurate (Rothenberg et al, 1996*b*).

Serum carbohydrate antigen 19–9 (CA 19–9), the sialylated Lewis[a] blood group antigen defined by the monoclonal antibody 1116 NS 19–9 (Koprowski et al, 1979), has been proved as the most sensitive and specific serum marker for pancreatic cancer (Pleskow et al, 1989; Rollhauser and Steinberg, 1998). The prognostic value of CA 19–9 for patients with pancreatic cancer treated with resection or radiotherapy is well established (Glenn et al, 1988; Katz et al, 1998; Rollhauser and Steinberg, 1998), but only few data regarding the prognostic value of CA 19–9 during chemotherapy with gemcitabine have been published so far.

*Received 7 July 1999*
*Revised 24 September 1999*
*Accepted 20 October 1999*

*Correspondence to:* U Halm

The aim of the study was therefore to assess the prognostic value of the tumour marker CA 19–9 in patients with advanced or metastatic pancreatic cancer treated with gemcitabine.

### PATIENTS AND METHODS

### Patients

Adult patients were included in this prospective study if they had metastatic or locally advanced, inoperable pancreatic cancer, a baseline Karnofsky performance status ≥ 60%, and histological diagnosis of ductal adenocarcinoma, which was obtained either percutaneously or during operation. Patients who had received previous chemotherapy or radiotherapy were excluded from the analysis. All patients received chemotherapy with gemcitabine as a single agent therapy as previously described (Rothenberg et al, 1996*a*; Burris et al, 1997). During the first cycle patients received gemcitabine in a dose of 1000 mg $m^{-2}$ once weekly for 7 weeks followed by 1 week of rest. Thereafter, gemcitabine was given once weekly for 3 weeks followed by 1 week of rest until progression of the disease. Progression was defined either by an increase of >25% of the longest perpendicular diameters of a mass lesion, the occurrence of a new lesion or malignant ascites, or a deterioration of performance status with inability of the patient to attend follow-up visits.

### Measurements

Serum samples for determination of the tumour marker CA 19–9 were obtained from every patient at baseline, after the first cycle (8

weeks) and thereafter before each cycle. In case of stent obstruction during chemotherapy, exchange of the stent was performed before serum samples were obtained. The CA 19–9 concentration was measured by a commercially available enzyme immunoassay (Enzymun-Test CA 19–9, Roche Diagnostics, Germany). The cut-off value given by the manufacturer was 22 U $l^{-1}$. The coefficient of variation in our laboratory was 7% ($n$ = 27). Taking the coefficient of variation into account, a fall of CA 19–9 during chemotherapy was defined as a decrease > 20% after the first cycle (8 weeks) of chemotherapy.

### Statistical analysis

Unadjusted median survival curves were constructed according to Kaplan and Meier (Kaplan and Meier, 1958). Differences in survival were calculated with the log-rank test and correlations with Spearman's rank correlation test. The proportional hazard multivariate model (Cox, 1972) was used to evaluate the relationship of differences of survival to patient and disease characteristics, which might influence the prognosis. Data showing significance or a trend for significance ($P < 0.20$) by univariate analysis were included in the multivariate model. A two-sided $P$-value < 0.05 was considered as statistically significant.

### RESULTS

The patient characteristics are listed in Table 1. Forty-three patients were consecutively included. The median age was 61 years (range 39–76 years), 20 patients were male and 23 female. Twenty-eight patients had evidence of metastatic disease and 15 of locally advanced disease. Prior to chemotherapy, eight patients with obstructive jaundice underwent endoscopic biliary prosthesis. Twelve patients had undergone palliative operation and seven patients had recurrent metastatic disease after previous Whipple's pancreatoduodenectomy. Most patients had moderately or poorly differentiated ductal adenocarcinoma. Seven patients (16%) were excluded from further analysis because no serial measurements could be performed. Five of these patients had normal CA 19–9 values at baseline and two were lost to follow-up. The 36 remaining patients were treated with a median cumulative dose of 15 g $m^{-2}$ (range 3–55 g $m^{-2}$). In 97% of the gemcitabine administrations doses were given on schedule. During 8 cycles a dose reduction was necessary due to haematological toxicity grade 3. The median baseline CA 19–9 concentration of the remaining 36 patients was 1515 U $l^{-1}$ (range 34–43 553 U $l^{-1}$).

**Table 1** Baseline patient characteristics

| Patient characteristics | |
|---|---|
| Age – years (range) | 61 (39–76) |
| Males/Females – *n* | 20/23 |
| Median baseline CA 19–9 – U $l^{-1}$ (range)[a] | 1515 (34–43553) |
| Tumour stage (UICC 1997) – *n* | |
| III/IVa | 15 |
| IVb | 28 |
| Tumour grading – *n* | |
| I | 4 |
| II | 26 |
| III | 12 |
| IV | 1 |
| Previous operation – *n* | |
| Whipple's pancreatoduodenectomy | 7 |
| Cholangiojejunostomy | 6 |
| Gastroenterostomy | 2 |
| Cholangiojejunostomy and gastroenterostomy | 4 |
| Biliary prosthesis – *n* | 8 |

[a] Patients with serial CA 19–9 measurements ($n$ = 36). Normal value of CA 19–9: < 22 U $l^{-1}$.

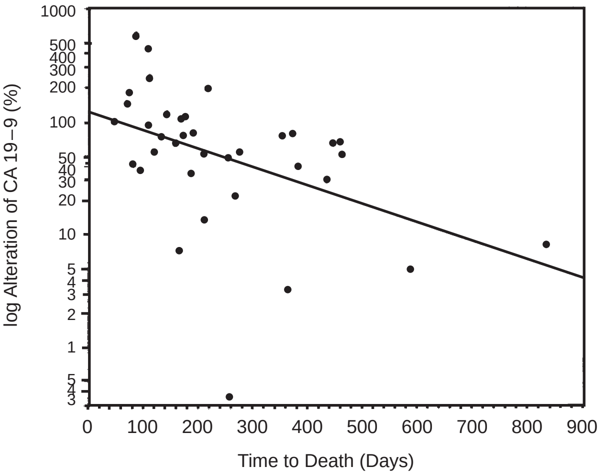

**Figure 1** Survival time in relation to the log alteration of CA 19–9 after 8 weeks of chemotherapy with gemcitabine ($r = -0.55$, $P = 0.001$)

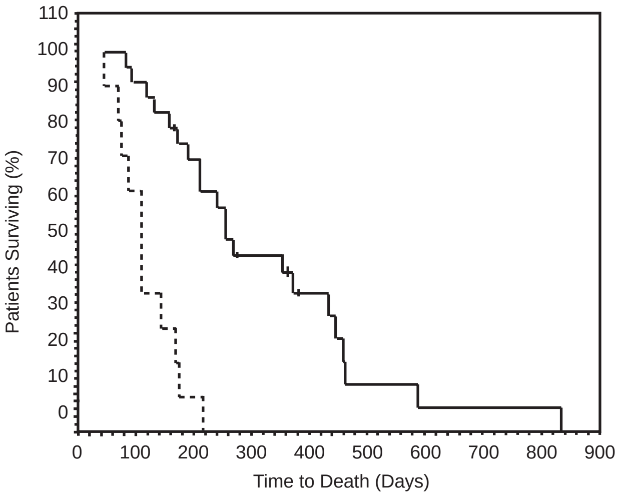

**Figure 2** Survival curves using the Kaplan–Meier method comparing the survival of patient with a CA 19–9 decrease > 20% (——) and ≤ 20% or a primary rise (-------) during the first 8 weeks of chemotherapy with gemcitabine. Patients with a CA 19–9 response > 20% had a significantly better median survival than patients with no response (268 vs 110 days; $P < 0.001$)

*© 2000 Cancer Research Campaign*

**Table 2** Possible prognostic factors identified by the Cox proportional hazards model

| Variable | Relative risk (95% confidence interval) | *P*-value |
|---|---|---|
| CA 19–9 response (> 20% vs. ≤ 20%) | 12.7 (4.3–38.1) | < 0.001 |
| Tumour grading (I/II vs. III/IV) | 3.4 (1.4–8.3) | 0.008 |
| Tumour stage (III/IVa vs. IVb) | 1.7 (0.7–4.1) | 0.25 |
| Baseline CA 19–9 concentration (quartiles) | 1.4 (1.0–2.0) | 0.04 |
| Prior palliative operation/Biliary stenting | 1.2 (0.8–1.9) | 0.35 |

A significant correlation between the percental alteration of CA 19–9 concentration and survival was found ($r = -0.55$; $P = 0.001$; Figure 1). Twenty-five (69%) of the 36 patients had a response of CA 19–9 to treatment with gemcitabine and 11 (31%) a progressive rise ($n = 9$) or a decrease of ≤20% ($n = 2$) of the CA 19–9 concentration. The median overall survival of the 36 patients with increased baseline CA 19–9 concentrations was 211 days (95% confidence interval (CI) 155–267 days). However, patients responding with CA 19–9 decrease > 20% had a median survival of 268 days (95% CI 123–413 days), which was significantly longer than the median survival of the patients who did not respond (110 days; 95% CI 83–137 days; $P < 0.001$; Figure 2). The median survival of patients with normal CA 19–9 concentrations at baseline was 168 days (95% CI 54–282). Similar results were obtained regarding time to progression. Patients responding with CA 19–9 > 20% had a significantly longer median time to progression (173 days; 95% CI 136–210 days) than patients who did not respond (75 days; 95% CI 41–109 days; $P < 0.001$).

After 8 weeks of chemotherapy, objective partial response was achieved in four patients (11%). All of them had a CA 19–9 decrease > 20%. Nineteen of the 25 patients with stable disease and two of seven with progressive disease after 8 weeks of treatment had decreasing CA 19–9 concentrations > 20%. Eighteen of the 25 patients responding with the tumour marker CA 19–9 > 20% experienced a recurrent increase. The median survival time from the beginning of recurrent rise to death was 96 days (95% CI 65–127 days).

Age and gender did not show significant or a trend for significant differences regarding survival by univariate analysis ($P > 0.20$). The alteration of CA 19–9 concentration, tumour stage and grading, previous operation, biliary stenting and the CA 19–9 concentration at baseline were included in the multivariate analysis. The Cox regression analysis showed the fall of CA 19–9 > 20% as the strongest independent factor for survival time. Tumour grading and the baseline CA 19–9 concentration, but neither surgical bypasses, biliary stenting, nor tumour stage had a significant influence on survival (Table 2).

### DISCUSSION

The prognosis of patients with locally advanced or metastatic pancreatic cancer is poor. All chemotherapeutic regimens used in the last decades failed to improve survival substantially (Ahlgren, 1996; Schnall and Macdonald, 1996). Recently, chemotherapy with gemcitabine has been shown for the first time to improve significantly both survival and quality of life in patients with advanced pancreatic cancer, although overall survival was poor and the majority of patients treated did not respond (Rothenberg et al, 1996*a*; Burris et al, 1997). The response of most solid tumours to chemotherapy is controlled by imaging techniques. A major problem of measuring tumour response during chemotherapy of pancreatic cancer is that computerized tomography, ultrasound, and other imaging techniques often fail to differentiate normal pancreas, local inflammation, and fibrosis from malignant tissue and therefore may be inaccurate in the assessment of the response (Rothenberg et al, 1996*b*). Consequently, the endpoints to assess tumour response were expanded by clinical parameters and quality of life, which has been proposed to be more appropriate than assessment of tumour diameters (Rothenberg et al, 1996*b*; Burris et al, 1997). However, these parameters are difficult to obtain and the results of the present study indicate that serial measurements of CA 19–9 concentrations during therapy with gemcitabine are useful to assess prognosis.

In accordance to previous studies, the overall survival in our study was poor with none of the patients surviving longer than 28 months (Rothenberg et al, 1996*b*; Burris et al, 1997). However, a fall of CA 19–9 > 20% after 8 weeks of chemotherapy was found to separate patients into groups with significantly different survival times. Moreover, not only the primary, but also a recurrent rise of CA 19–9 after an initial response was associated with a short median survival time of the patients. The presence of metastatic disease, high baseline tumour marker concentrations, and a poor performance status has been described as unfavourable prognostic factors (Lundin et al, 1994; Ishii et al, 1996). In contrast, in the multivariate analysis of our study, the alteration of CA 19–9 was the strongest independent prognostic factor. Since the median survival time of patients with a decrease of CA 19–9 ≤ 20% or a rise during the first cycles of chemotherapy or with a recurrent rise after initial response is very short, the measurement of CA 19–9 after 8 weeks of chemotherapy may together with other clinical parameters help to decide, whether further chemotherapy should be stopped.

Two of seven patients had falling CA 19–9 concentrations after 8 weeks of treatment despite disease progression. Similar results were obtained in a phase II trial treating patients with locally advanced or metastatic pancreatic cancer with gemcitabine, in which serial measurements were performed in 14 patients with evaluable disease. All patients with radiologically assessed partial response ($n = 2$) or stable disease ($n = 5$), but also four of seven patients with progressive disease exhibited a decrease of CA 19–9 during the course of chemotherapy (Carmichael et al, 1996). These inconsistent results remain difficult to interpret, but may be related to the inaccuracies in the assessment of tumour size.

The prognostic value of CA 19–9 has already been established in patients undergoing radiation or resection of pancreatic carcinoma. A better survival was found in patients responding with a falling CA 19–9 following radiotherapy (Katz et al, 1998; Okusaka et al, 1998). Increased CA 19–9 concentrations after resection of pancreatic cancer has been reported to be associated with poor survival compared with patients whose CA 19–9 had been normalized (Glenn et al, 1988; Tian et al, 1992; Safi et al, 1998). High preoperative concentrations of CA 19–9 correlate also with poor survival (Lundin et al, 1994; Safi et al, 1998). A decrease of > 15% of CA 19–9 concentrations during chemotherapy with 5-fluorouracil, epirubicin, and cisplatin has been shown to correlate with a better survival than a primary rise or a plateau of the CA 19–9 concentration. Patients with a decrease of > 15% of CA 19–9 had no progression of their disease as assessed by computerized tomography (Gogas et al, 1998). In contrast to our results, only 13 of 36 patients (36%) responded

*© 2000 Cancer Research Campaign* 

with a fall of CA 19–9 to the chemotherapy regimen. However, these results confirm that CA 19–9 helps to assess prognosis during chemotherapy of pancreatic cancer.

In conclusion, in patients with advanced or metastatic pancreatic cancer and increased baseline CA 19–9 concentrations, measurements of CA 19–9 should be performed after 8 weeks of treatment to assess prognosis together with other clinical parameters. In patients with an increase of CA 19–9 or with a decrease ≤ 20%, prognosis is extremely poor and with the exception of cases with significant improvement of performance status, further chemotherapy with gemcitabine seems of questionable value.

### ACKNOWLEDGEMENTS

Presented in part at the 1999 European Pancreatic Club meeting, Lüneburg, Germany. We thank Dr C Weisbrich at the Institute of Clinical Chemistry, University of Leipzig, for measurements of CA 19–9.

### REFERENCES

Aapro MS, Martin C and Hatty S (1998) Gemcitabine: a safety review. *Anticancer Drugs* **9**: 191–201

Ahlgren JD (1996) Chemotherapy for pancreatic carcinoma. *Cancer* **78**: 654–663

Burris HA, Moore MJ, Andersen J, Green MR, Rothenberg ML, Modiano MR, Cripps MC, Portenoy RK, Storniolo AM, Tarassoff P, Nelson R, Dorr FA, Stephens CD and Von Hoff DD (1997) Improvements in survival and clinical benefit with gemcitabine as first line therapy for patients with advanced pancreas cancer: a randomized trial. *J Clin Oncol* **15**: 2403–2413

Carmichael J, Fink U, Russel RCG, Spittle MF, Harris AL, Spiessi G and Blatter J (1996) Phase II study of gemcitabine in patients with advanced pancreatic cancer. *Br J Cancer* **73**: 101–105

Cox DR (1972) Regression models and life tables. *J R Stat Soc [B]* 34: 187–202

Glenn J, Steinberg WM, Kurtzman SH, Steinberg SM and Sindelar WF (1988) Evaluation of the utility of a radioimmunoassay for serum CA 19–9 levels in patients before and after treatment of carcinoma of the pancreas. *J Clin Oncol* **6**: 462–468

Gogas H, Lofts FJ, Evans TRJ, Daryanani S and Mansi JL (1998) Are serial measurements of CA 19–9 useful in predicting response to chemotherapy in patients with inoperable adenocarcinoma of the pancreas? *Br J Cancer* **77**: 325–328

Ishii H, Okada S, Nose H, Yoshimori M, Aoki K and Okusaka T (1996) Prognostic factors in patients with advanced pancreatic cancer treated with systemic chemotherapy. *Pancreas* **12**: 267–271

Kaplan EL and Meier P (1958) Non-parametric estimation from incomplete observations. *J Am Stat Assoc* **53**: 457–481

Katz A, Hanlon A, Lanciano R, Hoffman J and Coia L (1998) Prognostic value of CA 19–9 levels in patients with carcinoma of the pancreas treated with radiotherapy. *Int J Radiat Oncol Biol Phys* **41**: 393–396

Koprowski H, Steplewski Z, Mitchell K, Herlyn M, Herlyn D and Fulner P (1979) Colorectal carcinoma antigens detected by hybridoma antibodies. *Somatic Cell Genet* **5**: 957–971

Lundin J, Roberts PJ, Kuusela P and Haglund C (1994) The prognostic value of preoperative serum levels of CA 19–9 and CEA in patients with pancreatic cancer. *Br J Cancer* **69**: 515–519

Okusaka T, Okada S, Sato T, Wakasugi H, Saisho H, Furuse J, Ishikawa O, Matsuno S and Yokoyama S (1998) Tumor markers in evaluating the response to radiotherapy in unresectable pancreatic cancer. *Hepatogastroenterology* **45**: 867–872

Pleskow DK, Berger HJ, Gyves J, Allen E, McLean A and Podolsky DK (1989) Evaluation of a serologic marker, CA 19–9, in the diagnosis of pancreatic cancer. *Ann Intern Med* **110**: 704–709

Rollhauser C and Steinberg W (1998) Tumor antigens in pancreatic cancer. In *Pancreatic Cancer*, Reber HA (ed) pp 137–156. Humana Press: Totowa, New Jersey

Rothenberg ML, Moore MJ, Cripps MC, Andersen JS, Portenoy RK, Burris HA, Green MR, Tarassoff PG, Brown TD, Casper ES, Storniolo AM and Von Hoff DD (1996*a*) A phase II trial of gemcitabine in patients with 5-FU-refractory pancreas cancer. *Ann Oncol* **7**: 347–353

Rothenberg ML, Abbruzzese JL, Moore M, Portenoy RK, Robertson JM and Wanebo HJ (1996*b*) A rationale for expanding the endpoints for clinical trials in advanced pancreatic carcinoma. *Cancer* **78**: 627–632

Safi F, Schlosser W, Falkenreck S and Beger HG (1998) Prognostic value of CA 19–9 serum course in pancreatic cancer. *Hepatogastroenterology* 45: 253–259

Schnall SF and Macdonald JS (1996) Chemotherapy of adenocarcinoma of the pancreas. *Semin Oncol* **23**: 220–228

Tian F, Appert HE, Myles J and Howard JM (1992) Prognostic value of serum CA 19–9 levels in pancreatic adenocarcinoma. *Ann Surg* **215**: 350–355

*© 2000 Cancer Research Campaign*
